# Cephalometric and digital model analysis of dentoskeletal effects of infrazygomatic miniscrew vs. Essix- anchored Carriere Motion appliance for distalization of maxillary buccal segment: a randomized clinical trial

**DOI:** 10.1186/s12903-024-03925-3

**Published:** 2024-01-31

**Authors:** Eglal Ahmed Ghozy, Nehal Fouad Albelasy, Marwa Sameh Shamaa, Ahmed A. El-Bialy

**Affiliations:** Department of Orthodontics, Faculty of Dentistry, Mansoura University, Mansoura, 35116 Egypt

**Keywords:** Carriere motion appliance, Distalization, Class II malocclusion, Infrazygomatic miniscrews, Skeletal anchorage

## Abstract

**Trial design:**

Parallel.

**Objective:**

To compare skeletally anchored Carriere Motion appliance (CMA) for distalization of the maxillary buccal segment vs. Essix anchored CMA.

**Methods:**

Thirty-two class II malocclusion patients were randomly allocated into two equal groups. One group was treated with infrazygomatic (IZC) miniscrew- anchored CMA (IZCG) and the other group treated with Essix retainer- anchored CMA (EXG). Two lateral cephalograms and two digital models for upper and lower arches were taken for each patient: immediately before intervention and after distalization had been completed.

**Results:**

Distalization period was not significantly different between the two groups. In contrast to EXG, IZCG showed insignificant difference in ANB, lower incisor proclination, and mesial movement of the lower first molar. There was significant rotation with distal movement of maxillary canine and first molar in both groups.

**Conclusion:**

IZC anchored CMA could eliminate the side effects of class II elastics regarding lower incisor proclination, mesial movement lower molars with a more significant amount of distalization of the maxillary buccal segment but with significant molar rotation.

**Trial registration:**

The ClinicalTrials.gov Protocol Registration and Results System (PRS) has this RCT registered as (NCT05499221) on 12/08/2022.

## Background

One of the most frequent treatment challenges is Class II malocclusion, which accounts for almost a third of all malocclusions [1]. Carriere Motion appliance (CMA), (Henry Schein Orthodontics, CA, USA) was unveiled in 2004. By distalizing the entire maxillary buccal segment using class II elastics and mandibular anchors, CMA was designed to treat Class II molar relationship. The lingual arch, Essix retainer, or miniscrews are used as methods of anchorage to prevent protrusion of the mandibular incisors when the appliance is activated [2].

The application of the CMA has been assessed in former case reports [3–6]. One retrospective study [7] compared the use of CMA with full fixed orthodontic appliances in the mandibular arch vs. a lingual arch anchorage and found that both techniques caused lower incisors proclination. Other investigations [8–11] assessed the treatment by CMA anchored with Essix appliance and reported that the lower first molar moved and tipped mesially significantly with lower incisor proclination. Only one randomized clinical trial [12] (RCT) compared anchorage control using interdental miniscrews vs. Essix appliance and found that miniscrews reduced anchorage loss regarding the mandibular incisors.

The success of CMA, like that of the majority of orthodontic appliances, depends on the patient's commitment to intermaxillary elastics and lower retainer wear [9, 13]. It has the adverse consequences of class II elastics, such as proclination of the lower incisors and maxillary canine extrusion [8]. To avoid the negative effects of CMA with class II elastics, we can employ the CMA to distalize the maxillary buccal segment with intra-arch anchorage utilizing IZC miniscrews noting that they are contraindicated in cases of compromised immune defense, bleeding disorders, pathological bone quality, or inadequate oral hygiene [14, 15] and in children with deciduous or mixed dentition [15, 16].

To our knowledge, few studies have examined the effects of the CMA three-dimensionally [8, 10]. However, these assessments used cone beam computerized tomography (CBCT), which is not considered as a routine diagnostic tool owing to its high radiation exposure. Remarkably, only two studies [11, 17] have utilized models to examine the 3D effects of the CMA.

### Aim of the study

The objective of this study was to compare the 3D effects of IZC miniscrew anchored vs. Essix anchored CMA for distalization of the maxillary buccal segment using laterals cephalograms and digitized models.

## Methods

### Trial design

This is a parallel design RCT with a 1:1 allocation ratio. Patients were randomly allocated in the intervention and comparison group as follows:

a- IZC anchored CMA group (IZCG): IZC miniscrews were used for anchorage.
b- Essix anchored CMA group (EXG): Essix retainer in the lower arch was used for anchorage.

The ClinicalTrials.gov Protocol Registration and Results System (PRS) has this randomized clinical trial registered as (NCT05499221) on 12/08/2022.

### Ethics approval and consent to participate

The committee of research ethics in Mansoura University faculty of dentistry "Dental Research Ethics Committee" authorized this study. From January 2022 to January 2023, patients were enrolled from the outpatient clinic at the orthodontic department, Faculty of Dentistry, Mansoura University. All the parents of the enrolled patients signed the informed consent form as the patients were below the age of 16.

### Eligibility criteria

Patients aged (12–16) years with full permanent dentition and Class II malocclusion were included. Patients with systemic conditions, bad habits, transverse discrepancy, or previous orthodontic treatment were excluded.

### Intervention

The right size of CMA was selected in accordance with the manufacturer's recommendations. Then it was bonded to the upper canine and first molar.

**Fig. 1:**
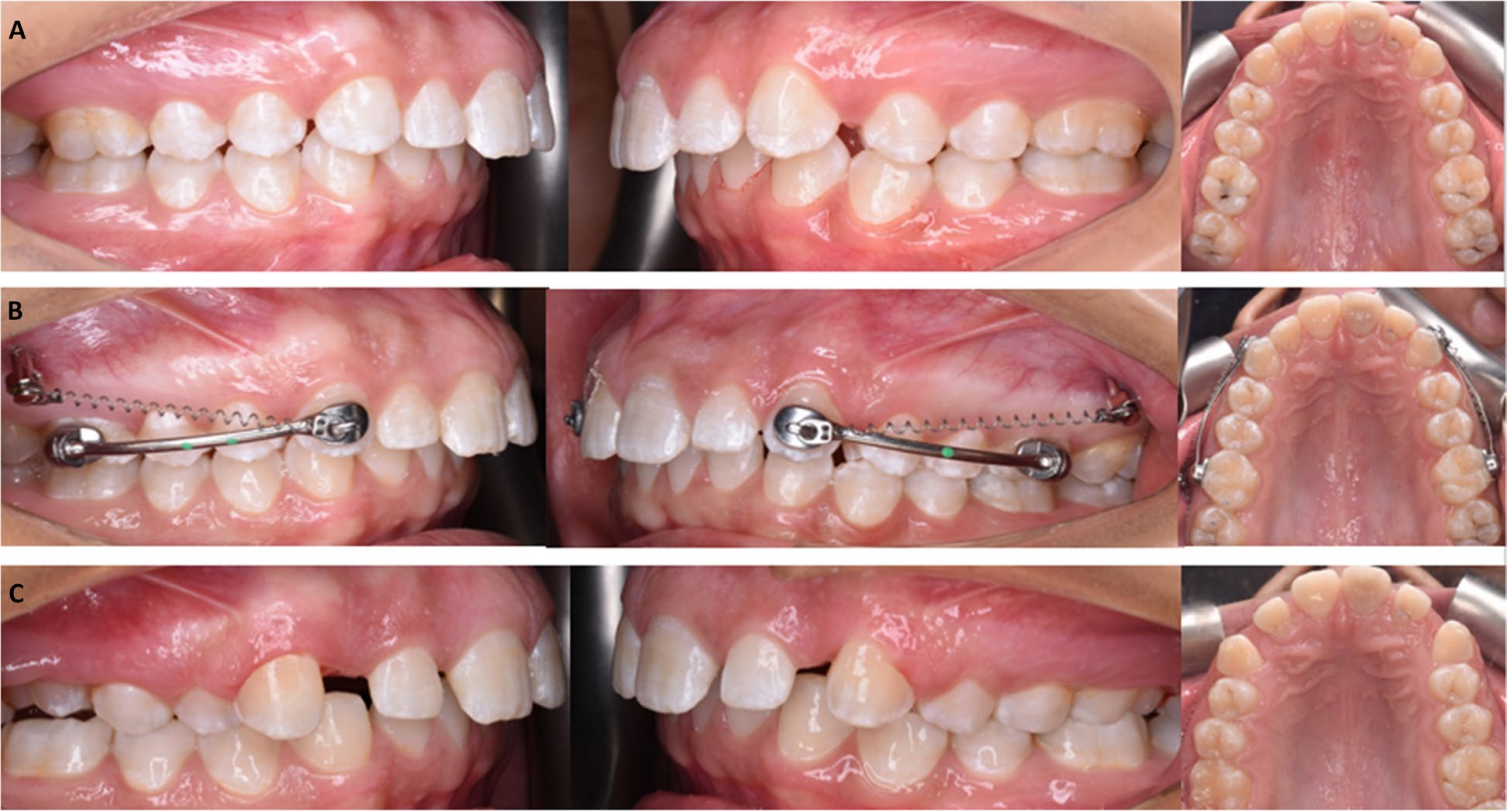
Intraoral photos of a case in IZCG; **A** predistalization, **B **Intervention, **C** postdistalization photos

In IZCG: two miniscrews (Bio-ray, New Taipei, Taiwan), 14 mm long and 2 mm in diameter, were placed in infrazygomatic crest area bilaterally and a closing coil spring was affixed bilaterally between the maxillary canine and the IZC miniscrews as shown in Fig. 1.

**Fig. 2:**
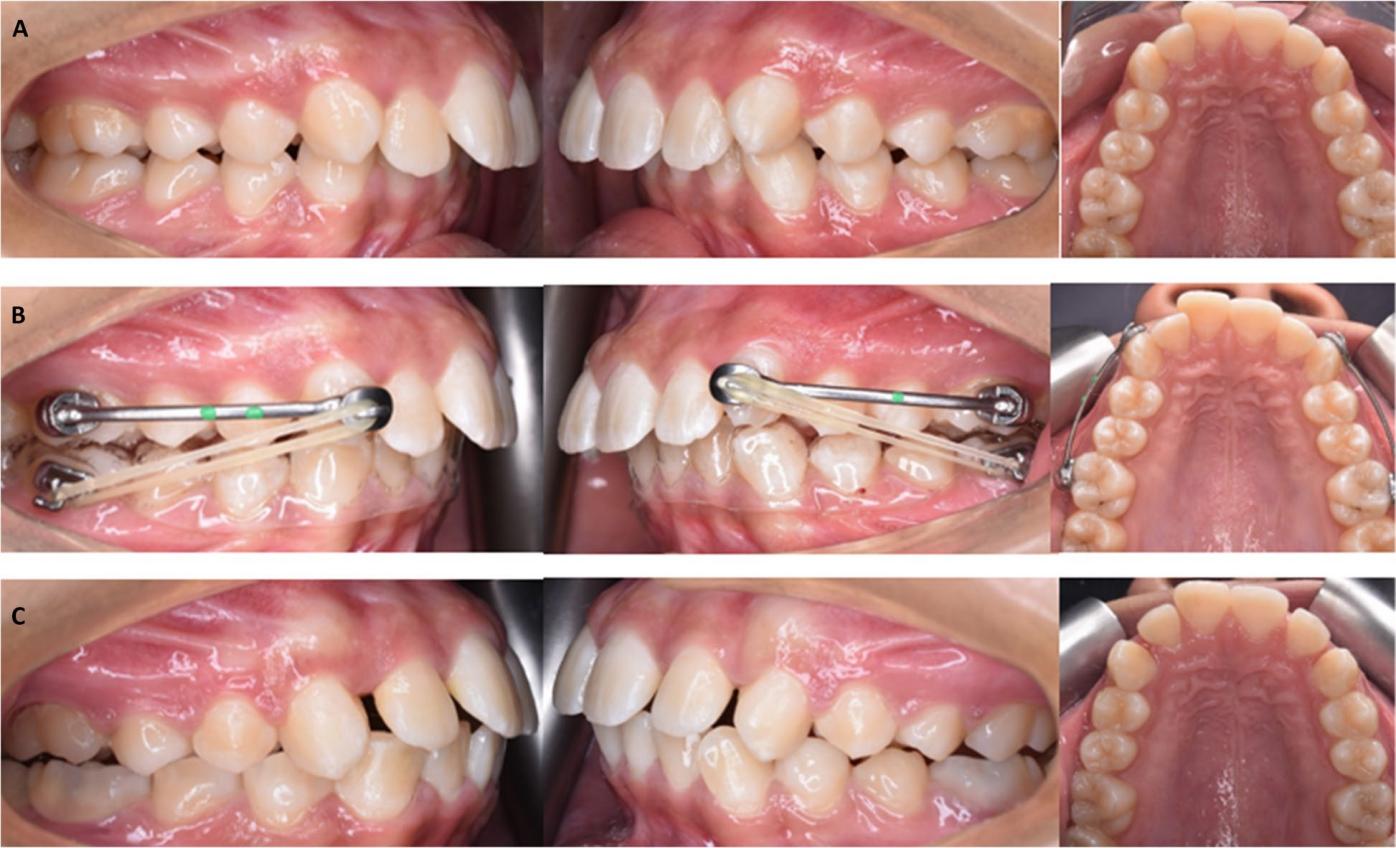
Intraoral photos of a case in EXG; **A** predistalization, **B** Intervention, **C** postdistalization photos

In EXG: After bonding buccal tubes to the lower first molars and taking an impression of the lower arch, a cast was made. The Essix appliance was made from a vacuum sheet of 1.5 mm thickness with a window around the buccal tubes for class II elastic attachment bilaterally as shown in Fig. 2. Heavy 1/4-inch elastics were utilized for the first month followed by heavy 3/16-inch elastics. Except during mealtimes, participants were told to wear the elastics day and night and to replace them every day.

Every four weeks, follow-up appointments were planned, and the appliance was debonded in both groups on reaching Class I relationship. Two lateral cephalograms were taken and two impressions for both upper and lower arches were obtained immediately before intervention and after completing distalization before starting the second phase of the treatment. Casts were made and scanned for obtaining digital models.

### Outcomes

**Fig. 3:**
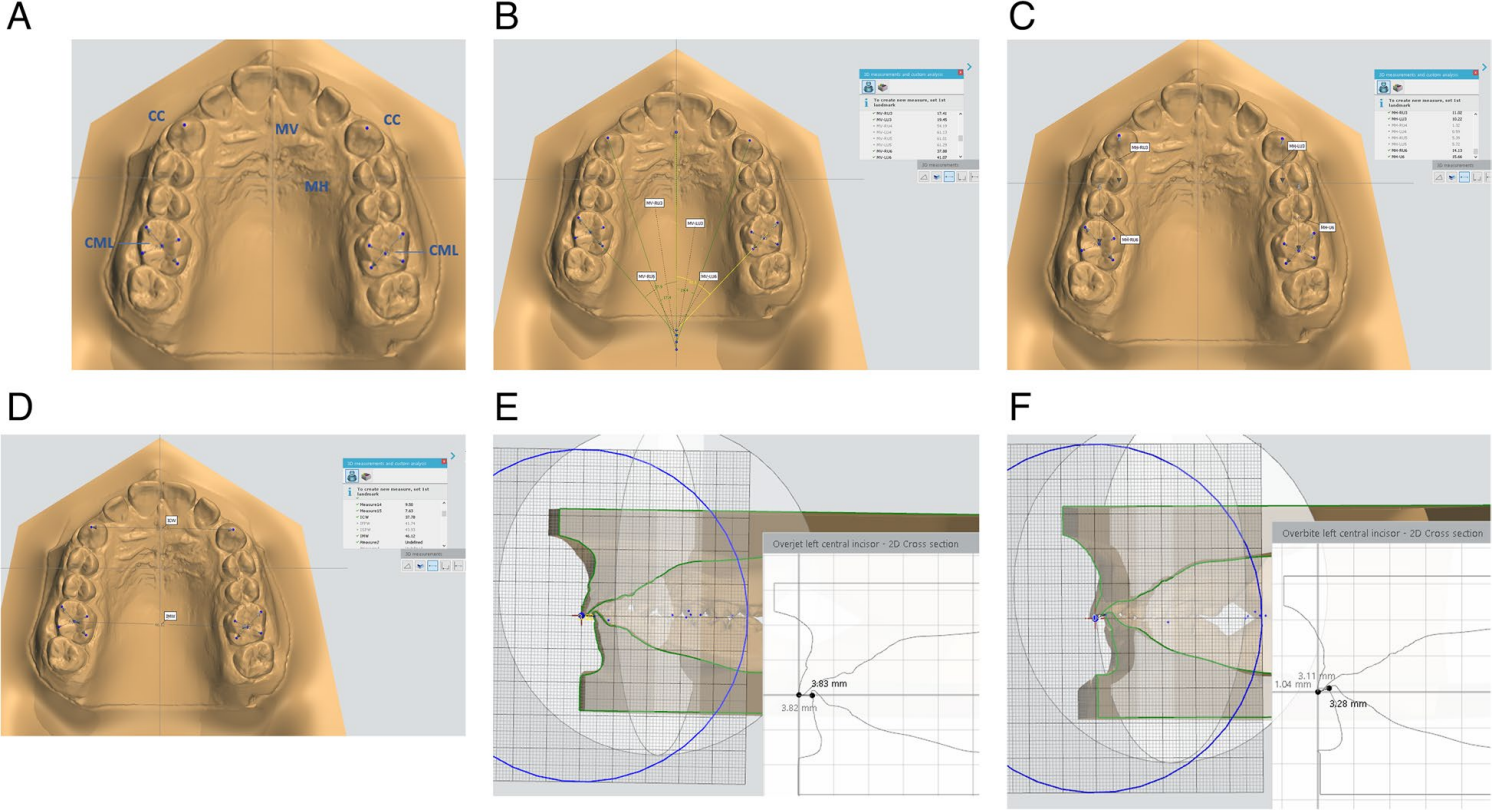
**A** Model landmarks. **B** Model angular measurements. **C** Model linear measurements. **D** Arch width. **E** Over jet measuring. **F** Overbite measuring on models

The primary outcomes were the treatment duration, skeletal and dental changes. A single, blinded assessor compared the de-identified cephalograms and the 3D digital models. The cephalograms were assessed using WebCeph (web-based program for cephalometric analysis). Table 1 shows the skeletal and dental measurements. The 3D model images were assessed using 3D measurements tool in OrthoAnalyzer software (3Shape, Copenhagen, Denmark) after model preparation and plane alignment by setting up the occlusal plane, the sagittal plane; MV line (A vertical reference plane drawn through the median palatine suture from the incisive papilla, perpendicular to the occlusal plane) and the coronal plane; MH line (the horizontal reference line passing through the left third rugae and perpendicular to MV). The model landmarks and measurements are described in detail in Tables 2 and 3 and illustrated in Fig. 3(A-F).

**Table 1:** Cephalometric measurements

**Skeletal measurements**	
SNA (°)	The angle between 3-point landmarks: S, N, and A point
SNB (°)	Sella-nasion to B point angle
ANB (°)	The angle between 3-point landmarks: A, N, B
**Vertical skeletal measurements**	
LAFH (mm.)	Lower anterior facial height (mm): distance between ANS and menton
PFH (mm.)	Posterior facial height (mm): distance between S and Gonion
**Dental measurements**	
U1-SN (°)	The angle measured between the long axis of the upper central incisor and the SN plane
U3 angle (°)	The angle measured between the long axis of the canine (cusp tip to root apex) and the Sella-Nasion line
U6 angle (°)	The angle measured between the long axis of the mesiobuccal cusp to the mesiobuccal root apex of the maxillary first molar and the Sella- Nasion line
L6 angle (°)	The angle measured between the long axis of the mesiobuccal cusp to the mesial root apex of the mandibular first molar and the Sella-Nasion line
IMPA (°)	The angle measured between the long axis of the mandibular central incisor and the Gonion-Menton line
Interincisal angle: IIA (°)	The angle measured between the long axis of the upper and lower central incisor
**Dental Linear measurements**	
U3 VP (mm.)	The vertical distance from the horizontal plane (SN-7) to the upper canine cusp tip
U6 VP (mm.)	The vertical distance from the horizontal plane (SN-7) to mesiobuccal cusp tip of the upper fisrt molar
L6m VP (mm.)	The vertical distance from the horizontal plane (SN-7) to the mesiobuccal cusp tip of the lower fisrt molar
L6d VP (mm.)	The vertical distance from the horizontal plane (SN-7) to the distobuccal cusp tip of the lower fisrt molar
L6 AP position (mm.)	The horizontal distance from the mesiobuccal cusp tip of the lower fisrt molar to the vertical plane (perpendicular to the horizontal plane (SN-7) from S point)

**Table 2:** Model landmarks (Fig. 3A)

cc	cusp tip of the maxillary canine.
mb	mesiobuccal cusp tip of the maxillary first molar.
mp	mesiopalatal cusp tip of the maxillary first molar.
db	distobuccal cusp tip of the maxillary first molar.
dp	distopalatal cusp tip of the maxillary first molar.
CMR	the point that bisects the mb- dp line with mp-db line on the maxillary right first molar.
CML	the point that bisects the mb-dp line with mp-db line on the maxillary left first molar.

**Table 3:** Model measurements

**Model angular measurements:** (Fig. 3B)	
MV- RU3	The angle between MV (A vertical reference line drawn through the median palatine suture from the incisive papilla) and the right canine cusp tip.
MV- LU3	The angle between MV and the left canine cusp tip.
MV- U3	Mean of MV-R3 and MV- L3
MV-RU6:	The angle between MV and mb-dp line of the right first molar.
MV-LU6:	The angle between MV and mb-dp line of the left first molar.
MV-U6:	Mean of MV-R6 and MV-L6
**Model linear measurements**: (Fig. 3C)	
MH- RU3	The perpendicular distance between upper right canine cusp tip and MH line (the horizontal reference line passing through the left third rugae and perpendicular to MV)
MH- LU3	The perpendicular distance between upper left canine cusp tip and MH line
MH- U3	Mean of MH-RU3 and MH- LU3
MH- RU6	The perpendicular distance between CMR and MH line
MH-LU6	The perpendicular distance between CML and MH line
MH-U6	Mean of MH- RU6 and MH- LU6
**Arch width: **(Fig. 3D)	
Inter-canine width (ICW)	distance between cc points of both right and left canines.
Inter-molar width (IMW)	distance between CMR and CML points.
Overjet (mm): Fig. 3E	measured from the labial surface of lower incisors to the incisal edge of upper incisors.
Overbite (mm): Fig. 3F	The amount of vertical ovelap of lower incisors.

The same assessor and a different observer assessed the pre and post intervention cephalometric images and the 3D cast images in order to statistically evaluate the intra- and interobserver reliability.

### Sample size calculation

Based on Fouda et al. study [12], using the G*Power software (version 3.1.9.7), and by applying the formula by Borm et al. [18], a sample size of 16 patients per group achieves 86.7% power with expected dropout rate of 20%.

### Randomization

Simple randomization was carried out via the randomization formula in Excel (Microsoft, Wash, USA). To ensure allocation concealment, the random numbers were printed, and the papers were folded and placed in a box. After bonding CMA, the participant chose a paper from the box then was allocated to the matching group.

### Masking

Single blinding: only the outcomes assessor was blinded, and data were sent blinded for statistical analysis. Lateral cephalometric radiographs and digital study models were de-identified prior to obtaining measurements. The appliances were not present at the time these records were obtained.

### Statistical analysis

Data were analyzed using IBM-SPSS software (Version 27.0. Armonk, NY: IBM Corp). Qualitative data were expressed as N (%). Quantitative data were initially tested for normality using Shapiro–Wilk’s test with data being normally distributed if *p* > 0.050 and were expressed as mean ± SD.

Chi-square, Fisher’s Exact, Fisher-Freeman-Halton Exact and paired-Samples t-tests were used for data comparison. The Independent-samples t-test was used to compare data between two groups. For any of the used tests, results were considered as statistically significant if *p* value ≤ 0.050.

## Results

### Participant flow

**Fig. 4:**
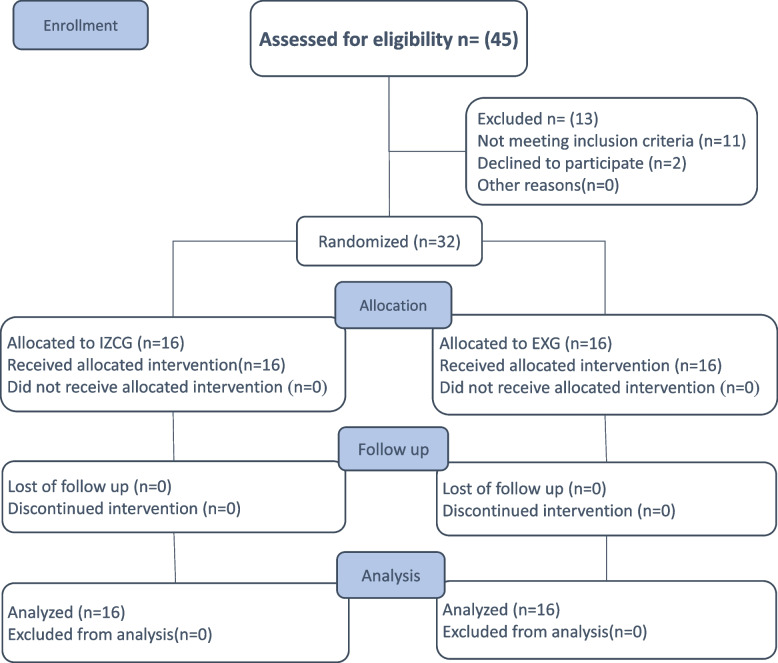
The Consolidated Standards of Reporting Trials (CONSORT) participant flow diagram

Recruitment initiated in January 2022 until January 2023. Thirty-two participants were recruited and randomized with a 1:1 ratio in either IZCG group (*n* = 16) or EXG group (*n* = 16). Distalization procedures were accomplished by June 2023 (Fig. 4).

### Baseline data

There was statistically insignificant difference between the two groups regarding male and female distribution inside the group and the mean age of patients as shown in Table 4.

**Table 4:** Clinical and Demographic data in IZCG vs. EXG

Characteristic	Group		Test of significance	
	**IZCG (*n* = 16)**	**EXG (*n* = 16)**		
**Categorical characteristic**	**N (%)**	**N (%)**	**χ**^**2**^	***p*-value**
**Sex**			.582	.446*
**Male**	4 (25%)	6 (37.5%)		
**Female**	12 (75%)	10 (62.5%)		
**Number of de-bonded CMAs**			-	.838**
**0**	11 (68.8%)	13 (81.3%)		
**1**	3 (18.8%)	1 (6.3%)		
**2**	2 (12.5%)	2 (12.5%)		
**Failure of anchorage device**			-	1.000***
**0 (no failure)**	13 (81.3%)	14 (87.5%)		
**1 (failure once)**	3 (18.8%)	2 (12.5%)		
**Numerical characteristic**	**Mean ± SD**	**Mean ± SD**	**t [30]**	***p*-value**
**Age (years)**	14.5 ± 1.4	13.8 ± 1.1	-1.563	.129^$^
**Treatment duration (months)**	6.3 ± 2.1	5.9 ± 2.8	-0.463	.647^$^

The tests of significance are ^*^chi-square test, ^**^Fisher-Freeman-Halton Exact Test, ^***^Fisher’s Exact test, and ^$^independent-samples t-test

### Outcomes measurements

#### Treatment duration

As illustrated in Table 4, CMA corrected class II molar relation in average duration of 6.3 ± 2.1 and 5.9 ± 2.8 in IZCG and EXG respectively. The difference in distalization duration between the two groups was insignificant. Only three out of the 32 infrazygomatic miniscrews that were inserted failed. On the other side, out of 16 Essix retainers, two broke before completing phase 1 of the treatment and needed to be remade.

### Skeletal and dental measurements

The comparison of pre and post intervention data in IZCG and EXG are shown in Tables 5 and 6. The comparison between the changes in IZCG vs. EXG is shown in Table 7.

**Table 5:** Comparisons of pre-post data in IZCG group

Characteristic	Pre	Post	Difference	95% CI		*p*-value	Cohen’s d
				Lower bound	Upper bound		
**SNA (°)**	83.8 ± 3.9	83.8 ± 4	0 ± 0.3	-0.2	0.2	.949	0.016
**SNB (°)**	78.5 ± 4	78.7 ± 4.2	0.2 ± 0.8	-0.2	0.6	.329	0.252
**ANB (°)**	5.2 ± 1.4	5.1 ± 1.7	-0.1 ± 0.7	-0.5	0.2	.452	0.193
**LAFH (mm)**	61.1 ± 4.2	61.1 ± 4.2	0 ± 1.4	-0.7	0.8	.931	0.022
**PFH (mm)**	71.3 ± 6.2	71.4 ± 6.3	0.1 ± 1	-0.5	0.6	.743	0.083
**U1-SN (°)**	104.2 ± 11.2	100.4 ± 11.3	-3.8 ± 2	-4.9	-2.8	** < .001**	1.931
**U3 ANGLE (°)**	97.6 ± 7.2	90.8 ± 8.2	-6.8 ± 4.9	-9.4	-4.1	** < .001**	1.387
**U6 ANGLE (°)**	75.1 ± 8.1	69.3 ± 9.5	-5.8 ± 4.2	-8.1	-3.5	** < .001**	1.370
**L6 ANGLE (°)**	59.8 ± 8.6	60.1 ± 8.4	0.4 ± 0.8	0	0.8	.071	0.485
**IMPA (°)**	95.7 ± 5.8	95.5 ± 6.1	-0.2 ± 0.7	-0.6	0.2	.246	0.302
**IIA (°)**	127.5 ± 11.5	131 ± 11.6	3.4 ± 2.6	2.1	4.8	** < .001**	1.330
**U3 VP (mm)**	64.8 ± 3.2	64.3 ± 3.3	-0.5 ± 0.8	-0.9	-0.1	**.030**	0.598
**U6 VP (mm)**	61.4 ± 2.5	59.7 ± 2.9	-1.7 ± 1.3	-2.4	-1.1	** < .001**	1.365
**L6m VP (mm)**	63 ± 2.9	63 ± 2.7	0 ± 0.5	-0.2	0.3	.839	0.052
**L6d VP (mm)**	61.3 ± 3	61.2 ± 2.9	0 ± 0.5	-0.3	0.2	.634	0.121
**L6 AP (mm)**	39.3 ± 6.5	39.2 ± 6.5	0 ± 0.2	-0.2	0.1	.267	0.288
**Model measurements**							
**MV- U3**	36.8 ± 5.4	50.3 ± 6.6	13.5 ± 5.9	10.4	16.7	** < .001**	2.287
**MV- U6**	31.2 ± 6.2	46.9 ± 7.1	15.6 ± 7	11.9	19.3	** < .001**	2.233
**MH- U3**	12 ± 2.5	8.9 ± 2.3	-3.1 ± 0.9	-3.6	-2.6	** < .001**	3.453
**MH- U6**	12.1 ± 2.2	15.4 ± 1.9	3.2 ± 1.2	2.6	3.9	** < .001**	2.691
**ICW**	33.9 ± 2.4	44.6 ± 2.8	10.7 ± 3	9.1	12.3	** < .001**	3.584
**IMW**	45.4 ± 2.9	46.3 ± 3.1	0.9 ± 0.8	0.5	1.4	** < .001**	1.123
**OJ**	4.9 ± 1.7	3.3 ± 1.5	-1.6 ± 1	-2.1	-1	** < .001**	1.525
**OB**	3 ± 1.7	3 ± 1.7	0 ± 0.8	-0.4	0.4	.975	0.008

Data is expressed as mean ± SD. The test of significance is Paired-Samples t-test. Effect size is presented as Cohen’s d (effect size is considered as small, medium, and large if Cohen’s d = 0.2, 0.5, and 0.8, respectively)

**Table 6:** Comparisons of pre-post data in EXG group

**Characteristic**	**Pre**	**Post**	**Difference**	**95% CI**		***p*-value**	**Cohen’s d**
				Lower bound	Upper bound		
**SNA (°)**	81.4 ± 3.2	80.9 ± 3	-0.5 ± 0.7	-0.9	-0.1	**.012**	0.710
**SNB (°)**	75.4 ± 2.6	76.3 ± 2.6	0.9 ± 0.5	0.6	1.1	** < .001**	1.756
**ANB (°)**	5.9 ± 1.5	4.6 ± 1.3	-1.3 ± 1.1	-1.9	-0.7	** < .001**	1.156
**LAFH (mm)**	62.3 ± 6.5	63.4 ± 6.4	1.1 ± 0.8	0.7	1.6	** < .001**	1.344
**PFH (mm)**	67.5 ± 6	68.9 ± 5.9	1.4 ± 0.9	0.9	1.9	** < .001**	1.510
**U1-SN (°)**	100.6 ± 6.3	99.3 ± 6	-1.4 ± 0.8	-1.8	-0.9	** < .001**	1.630
**U3 ANGLE (°)**	90.5 ± 5.6	85.1 ± 5	-5.4 ± 3.7	-7.3	-3.4	** < .001**	1.478
**U6 ANGLE (°)**	69.7 ± 3.1	61.6 ± 3	-8.1 ± 2.7	-9.6	-6.7	** < .001**	3.030
**L6 ANGLE (°)**	58.7 ± 5.2	53.5 ± 5.5	-5.2 ± 3.4	-7.1	-3.3	** < .001**	1.512
**IMPA (°)**	97.6 ± 5.4	101.2 ± 5.4	3.5 ± 1.3	2.8	4.2	** < .001**	2.632
**IIA (°)**	125.3 ± 9.6	121.4 ± 10.4	-3.9 ± 2.8	-5.4	-2.4	** < .001**	1.370
**U3 VP (mm)**	64.4 ± 4.7	67.4 ± 4.4	3 ± 2	2	4.1	** < .001**	1.523
**U6 VP (mm)**	60 ± 4.5	61.2 ± 4.7	1.2 ± 0.9	0.7	1.7	** < .001**	1.341
**L6m VP (mm)**	63.8 ± 4.7	62.3 ± 4.9	-1.6 ± 0.8	-2	-1.2	** < .001**	1.987
**L6d VP (mm)**	61.5 ± 4.7	60.2 ± 4.9	-1.3 ± 0.9	-1.7	-0.8	** < .001**	1.361
**L6 AP (mm)**	35.5 ± 4.6	37.2 ± 4.6	1.6 ± 1.2	1	2.3	** < .001**	1.309
**Model measurements**							
**MV- U3**	37 ± 8	40.8 ± 8	3.8 ± 1	3.2	4.4	** < .001**	3.635
**MV- U6**	30 ± 5.4	37.8 ± 7.3	7.7 ± 3.2	6	9.5	** < .001**	2.397
**MH- U3**	10.7 ± 1.9	8.4 ± 2	-2.3 ± 0.7	-2.7	-2	** < .001**	3.497
**MH- U6**	13.9 ± 1.5	16.1 ± 1.5	2.2 ± 0.8	1.8	2.6	** < .001**	2.804
**ICW**	33.6 ± 3	35.9 ± 3.3	2..3 ± 0.7	1.9	2.7	** < .001**	3.227
**IMW**	45.4 ± 2.8	45.3 ± 3	-0.1 ± 0.9	-0.5	0.3	.565	0.147
**OJ**	4.7 ± 1.4	3.3 ± 1.2	-1.4 ± 0.9	-1.8	-0.9	** < .001**	1.622
**OB**	3.7 ± 0.8	2.4 ± 1.1	-1.3 ± 0.9	-1.8	-0.8	** < .001**	1.393

Data is expressed as mean ± SD. The test of significance is Paired-Samples t-test. Effect size is presented as Cohen’s d (effect size is considered as small, medium, and large if Cohen’s d = 0.2, 0.5, and 0.8, respectively)

**Table 7:** Comparisons of pre-post change in IZCG vs. EXG

**Characteristic**	**IZCG**	**EXG**	**95% CI**		***p*-value**	**Cohen’s d**
			Lower bound	Upper bound		
**SNA (°)**	0 ± 0.3	-0.5 ± 0.7	0.1	0.9	**.019**	0.879
**SNB (°)**	0.2 ± 0.8	0.9 ± 0.5	-1.1	-0.2	**.008**	1.004
**ANB (°)**	-0.1 ± 0.7	-1.3 ± 1.1	0.5	1.9	**.001**	1.240
**LAFH (mm)**	0 ± 1.4	1.1 ± 0.8	-1.9	-0.3	**.012**	.946
**PFH (mm)**	0.1 ± 1	1.4 ± 0.9	-2	-0.6	**.001**	1.334
**U1-SN (°)**	-3.8 ± 2	-1.4 ± 0.8	-3.6	-1.3	** < .001**	1.618
**U3 ANGLE (°)**	-6.8 ± 4.9	-5.4 ± 3.7	-4.5	1.7	.378	0.317
**U6 ANGLE (°)**	-5.8 ± 4.2	-8.1 ± 2.7	-0.2	4.9	.073	0.657
**L6 ANGLE (°)**	0.4 ± 0.8	-5.2 ± 3.4	3.7	7.5	** < .001**	2.236
**IMPA (°)**	-0.2 ± 0.7	3.5 ± 1.3	-4.5	-3	** < .001**	3.512
**IIA (°)**	3.4 ± 2.6	-3.9 ± 2.8	5.4	9.3	** < .001**	2.699
**U3 VP (mm)**	-0.5 ± 0.8	3 ± 2	-4.6	-2.4	** < .001**	2.314
**U6 VP (mm)**	-1.7 ± 1.3	1.2 ± 0.9	-3.7	-2.1	.** < .001**	2.671
**L6m VP (mm)**	0 ± 0.5	-1.6 ± 0.8	1.1	2.1	** < .001**	2.443
**L6d VP (mm)**	0 ± 0.5	1.3 ± 0.9	0.7	1.7	** < .001**	1.682
**L6 AP (mm)**	-0.1 ± 0.2	1.6 ± 1.2	-2.3	-1	** < .001**	1.846
**Model measurements**						
**MV- U3**	13.5 ± 5.9	3.8 ± 1	6.5	12.9	** < .001**	2.286
**MV- U6**	15.6 ± 7	7.7 ± 3.2	3.9	11.9	** < .001**	1.448
**MH- U3**	-3.1 ± 0.9	-2.3 ± 0.7	-1.3	-0.2	.**010**	0.967
**MH- U6**	3.2 ± 1.2	2.2 ± 0.8	0.3	1.8	**.006**	1.045
**ICW**	10.7 ± 3	2..3 ± 0.7	6.8	10	** < .001**	3.868
**IMW**	0.9 ± 0.8	-0.1 ± 0.9	.04	1.6	**.001**	1.251
**OJ**	-1.6 ± 1	-1.4 ± 0.9	-0.9	0.5	.622	0.176
**OB**	0 ± 0.8	-1.3 ± 0.9	0.7	1.9	** < .001**	1.508

Data is expressed as mean ± SD. The test of significance is Independent-Samples t-test. Effect size is presented as Cohen’s d (effect size is considered as small, medium, and large if Cohen’s d = 0.2, 0.5, and 0.8, respectively)

### Reliability testing

Intraclass correlation coefficient (ICC) was used for intra-, and inter-observer absolute agreement in 16 participants. There was excellent intra- (0.985), and inter-observer (0.981) absolute agreement.

### Harms

Apart from the discomfort experienced by some patients who received the miniscrews, no substantial hazards were seen during the trial.

## Discussion

The idea of CMA is to distalize the entire posterior maxillary segment using class II elastics and mandibular anchoring, correcting Class II molar relationship. No previous RCT evaluated CMA compared to IZC- anchored one. So, the aim of this RCT was to compare distalization of the maxillary buccal segment using skeletaly anchored CMA vs. conventionally anchored CMA.

There are few studies which analyzed the 3D effects of the CMA using CBCT, which is not a routine orthodontic record owing to the excess radiation exposure [8, 10]. Interestingly, there are few studies that used models to study the 3D effects of the CMA [11, 17]. Since this study is one in a few investigations of digital models, only the results of the cephalometric radiographs are comparable to other studies.

### Treatment duration

The CMA corrected the molar relation class II in average duration of 6.3 and 5.9 months in IZCG and EXG respectively with insignificant difference between the two groups. The average distalization time of both groups was similar to that found in Yin et al. study [13] (6.3 months), but longer than that found in other researches [7–10, 19]. This might be because the majority of the participants in this study were older with higher bone densities. However, compared to skeletal anchorage distalization appliances and conventional ones, the distalization period was shorter (8.2 and 8 months, respectively) [20].

### Skeletal effects

In IZCG, there were insignificant skeletal sagittal and vertical changes. This is consistent with the majority of publications which claimed that intraoral maxillary distalizers only had slight indirect skeletal effects with direct dentoalveolar changes [21, 22]. While in EXG, there was significant sagittal changes. These findings in EXG were similar to previous studies [17, 19]. However, other studies [7, 9, 10, 12, 13] found insignificant sagittal changes during the treatment with CMA and attributed that to the more dentoalveolar effects of class II elastics [23]. Similar to other studies [7, 9, 12, 13, 19], significant increase of LAFH and PFH was found in EXG. This was due to the extrusion of lower first molars, the distal tipping and extrusion of upper molars by class II elastics in EXG.

### Dental effects

In EXG, there was a significant increase in the lower incisor proclination (3.5° ± 1.3) and the lower first molar moved mesial with significant mesial tipping and significant extrusion. On the other hand, these findings were not found in IZCG because no class II elastics were used. The horizontal and vertical components of the forces exerted by class II elastics explain the mesialization and extrusion of the lower molars in EXG denoting that molar class II correction is an integration of mandibular molar mesialization and maxillary molar distalization. Transferring the anchorage control in the mandibular arch to the maxillary one by using IZC miniscrews aided in correcting class II molar relation by maxillary distalization only without affecting the lower arch. Despite the fact that Fouda et al. [12] used miniscrews in the lower arch, they did not prevent the mesial movement of lower second molar. Different studies also found significant mesial movement, tipping, and extrusion of lower first molars with CMA [7, 8, 10, 17]. In several previous studies, with the use of CMA, lower incisor proclination was a noticeable result [7–10, 12, 13, 17, 19].

Maxillary canine and first molar were significantly distalized by -3.1 ± 0.9, and 3.2 ± 1.2 mm respectively in IZCG. While in EXG, they were significantly distalized by -2.3 ± 0.7 and 2.2 ± 0.8 mm respectively. The extent of distalization was significantly higher in IZCG compared to EXG. The amount of distalization of upper first molar was comparable to that reported in earlier research [7, 8, 10, 12, 13, 17]. The distalization of the entire maxillary buccal segment by CMA means that there was no anchorage loss in the premolar area unlike other distalizers that required retraction of the premolars and canines after molar distalization [24, 25].

This RCT found significant distal tipping of maxillary molar. So, it can be said that the ball-and-socket joint partially reduced molar tipping but did not eliminate it entirely as claimed [2]. Distal tipping of the upper molars was also reported in some other previous studies [7, 8, 10, 12, 13]. Additionally, distal tipping of the upper canine was detected in some previous studies [8, 10, 12] which is against the claims that the CMA's front section is a rigid half-round arm that controls the canine's inclination permitting bodily movement [2].

The significant intrusion of upper canine and first molar in IZCG and the significant extrusion of them in EXG can be attributed to the vertical force component of the Class II elastics in EXG which is the opposite in IZCG. Regarding the upper canine extrusion, some earlier studies showed similar results [8, 10, 12]. Regarding the upper first molar extrusion, a former study showed similar results [10].

The significant rotation of upper canine and upper first molar in both IZCG and EXG is due to the ball and socket joint in the molar pad [2]. Therefore, CMA corrected Class II partially by distal derotation with distalization of the maxillary first molars. Previous studies [8, 10] also proved upper molar and canine rotation but using CBCT.

The upper incisor inclination decreased more significantly in IZCG than in EXG. So, unlike other distalizers, CMA did not cause anchorage loss in the maxillary anterior segment. This decrease could be due to the spontaneous movement of the incisors in the space created by the distalization. However, some other studies found a slight proclination of the maxillary incisors resulting from the proclination of lower incisors [7, 8, 10, 13]. In contrast, other earlier studies [9, 17] noted that CMA did not affect the maxillary incisors because CMA distalized the maxillary buccal segment without being attached to the maxillary incisors.

The overjet decreased significantly in both IZCG and EXG by 1.6 ± 1 and 1.4 ± 0.9 mm respectively. But the change between the two groups was not statistically different. In IZCG the decrease in overjet can be due to the spontaneous distal movement of incisors into the space created after the distalization of the buccal segment. In EXG, the overjet was notably reduced by mandibular incisors proclination like what was found in former studies [7–10, 13]

In IZCG there was no significant change in the overbite. On the other hand, in EXG the overbite decreased significantly because class II elastics caused extrusion of lower first molars and upper first molars, distal tipping of upper molars and flaring of the lower incisors. In earlier research, CMA also significantly decreased the overbite [7–10, 13]

There was a more significant increase in the ICW in IZCG than EXG. While IMW increased only in IZCG. Hermann et al. [17] reported nearly no differences in anterior and posterior dental arch width and intercanine distance before and after CMA. However, they found little buccal movement of the canine and first molar [17].

### Limitations

The patients and the operator in this trial could not be blinded to the treatment modality. No treatment was finished at the time of data collection. There was no report on patient compliance.

### Generalizability

This study's generalizability might be constrained as it only involved one dental facility and one Phd candidate performing the treatments on only one ethnic group was investigated.

## Conclusion

IZC anchored CMA resulted in a more significant distalization of the maxillary buccal segment than the Essix anchored one with no significant difference between them regarding the duration of distalization. However, it was not bodily distalization, due to the significant molar rotation. IZC anchored CMA eliminated the negative effects of class II elastics on lower incisor inclination, mesial movement, tipping, and extrusion of lower molars, with no significant effect on the lower face height indicating that transferring the anchorage to the maxillary arch by using IZC miniscrews could correct class II malocclusion by only distalization without any effect on the lower arch.

## Data Availability

All the datasets used and analyzed during the current study are available from the corresponding author on reasonable request.

## Abbreviations

CMA: Carriere motion appliance
IZC: Infrazygomatic crest
IZCG: Infrazygomatic-miniscrews anchored CMA group
EXG: Essix- anchored CMA group

## References

[1] Moyers RE, Riolo ML, Guire KE, Wainright RL, Bookstein FL. Differential diagnosis of Class II malocclusions: Part 1. Facial types associated with Class II malocclusions. Am J Orthod. 1980;78(5):477–94. 10.1016/0002-9416(80)90299-7 6933855

[2] Carrière L (2004). A new Class II distalizer. J Clin Orthod.

[3] de Carlos VF (2006). Distalizer treatment of an adult Class II, division 2 malocclusion. J Clin Orthod.

[4] Schupp W, Haubrich J, Neumann I (2010). Class II correction with the Invisalign system. J Clin Orthod.

[5] Fouda AS, Aboulfotouh MH, Attia KH, Abouelezz A (2020). Carriere Motion Appliance with miniscrew anchorage for treatment of Class II, division 1 malocclusion. J Clin Orthod.

[6] Lombardo L, Cremonini F, Oliverio T, Cervinara F, Siciliani G (2022). Class II correction with Carriere Motion 3D Appliance and clear aligner therapy. J Clin Orthod.

[7] Sandifer CL, English JD, Colville CD, Gallerano RL, Akyalcin S (2014). Treatment effects of the Carrière distalizer using lingual arch and full fixed appliances. J World Fed Orthod.

[8] Areepong D, Kim KB, Oliver DR, Ueno H (2020). The Class II Carriere Motion appliance: A 3D CBCT evaluation of the effects on the dentition. Angle Orthod.

[9] Kim-Berman H, McNamara JA, Lints JP, McMullen C, Franchi L (2019). Treatment effects of the Carriere® Motion 3D™ appliance for the correction of Class II malocclusion in adolescents. Angle Orthod.

[10] Wilson B, Konstantoni N, Kim KB, Foley P, Ueno H (2021). Three-dimensional cone-beam computed tomography comparison of shorty and standard Class II Carriere Motion appliance. Angle Orthod.

[11] Nercellas Rodríguez AR, Colino Gallardo P, Zubizarreta-Macho Á, ColinoPaniagua C, Alvarado Lorenzo A, Albaladejo MA (2023). A New Digital Method to Quantify the Effects Produced by Carriere Motion Appliance. J Pers Med.

[12] Fouda AS, Attia KH, Abouelezz AM, El-Ghafour MA, Aboulfotouh MH (2022). Anchorage control using miniscrews in comparison to Essix appliance in treatment of postpubertal patients with Class II malocclusion using Carriere Motion Appliance: A randomized clinical trial. Angle Orthod.

[13] Yin K, Han E, Guo J, Yasumura T, Grauer D, Sameshima G (2019). Evaluating the treatment effectiveness and efficiency of Carriere Distalizer: a cephalometric and study model comparison of Class II appliances. Prog Orthod.

[14] Chen YJ, Chang HH, Huang CY, Hung HC, Lai EHH, Yao CCJ (2007). A retrospective analysis of the failure rate of three different orthodontic skeletal anchorage systems. Clin Oral Implants Res.

[15] Cornelius C-P, Ehrenfeld M (2010). The use of MMF screws: surgical technique, indications, contraindications, and common problems in review of the literature. Craniomaxillofac Trauma Reconstr.

[16] Chang H-P, Tseng Y-C (2014). Miniscrew implant applications in contemporary orthodontics. Kaohsiung J Med Sci.

[17] Schmid-Herrmann CU, Delfs J, Mahaini L, Schumacher E, Hirsch C, Koehne T (2023). Retrospective investigation of the 3D effects of the Carriere Motion 3D appliance using model and cephalometric superimposition. Clin Oral Investig.

[18] Borm GF, Fransen J, Lemmens WA (2007). A simple sample size formula for analysis of covariance in randomized clinical trials. J Clin Epidemiol.

[19] Luca L, Francesca C, Daniela G, Alfredo SG, Giuseppe S (2022). Cephalometric analysis of dental and skeletal effects of Carriere Motion 3D appliance for Class II malocclusion. Am J Orthod Dentofac Orthop.

[20] Soheilifar S, Mohebi S, Ameli N (2019). Maxillary molar distalization using conventional versus skeletal anchorage devices: a systematic review and meta-analysis. Int Orthod.

[21] Gulati S, Kharbanda O, Parkash H (1998). Dental and skeletal changes after intraoral molar distalization with sectional jig assembly. Am J Orthod Dentofac Orthop.

[22] Fontana M, Cozzani M, Caprioglio A (2012). Non-compliance maxillary molar distalizing appliances: an overview of the last decade. Prog Orthod.

[23] Janson G, Sathler R, Fernandes TMF, Branco NCC, de Freitas MR (2013). Correction of Class II malocclusion with Class II elastics: a systematic review. Am J Orthod Dentofac Orthop.

[24] Bolla E, Muratore F, Carano A, Bowman SJ (2002). Evaluation of maxillary molar distalization with the distal jet: a comparison with other contemporary methods. Angle Orthod.

[25] Carano A (1996). The distal jet for upper molar distalization. J Clin Orthod.

